# Tissue Infiltrating LTi—Like Group 3 Innate Lymphoid Cells and T Follicular Helper Cells in Graves' and Hashimoto's Thyroiditis

**DOI:** 10.3389/fimmu.2020.00601

**Published:** 2020-04-09

**Authors:** Audrey Mohr, Christophe Trésallet, Natacha Monot, Adeline Bauvois, Delphine Abiven, Muhammad Atif, Laetitia Claër, Rajneesh Malhotra, Gaëll Mayer, Robert Balderas, Outi Vaarala, Gabrielle Deniziaut, Isabelle Brocheriou, Camille Buffet, Laurence Leenhardt, Guy Gorochov, Makoto Miyara

**Affiliations:** ^1^Sorbonne Université, Inserm, Centre d'immunologie et des maladies infectieuses—Paris (CIMI-PARIS), Paris, France; ^2^Service de Chirurgie Digestive, Bariatrique et Endocrinienne, Hôpital Avicenne, Bobigny, France; ^3^Université Paris Nord Seine St Denis Paris 13, Laboratoire d'imagerie Biomédicale (LIB) INSERM CNRS U678, CHU Pitié-Salpêtriẽre, Paris, France; ^4^Sorbonne Université, Inserm, Centre d'immunologie et des maladies infectieuses—Paris (CIMI-PARIS), AP-HP Hôpital Pitié-Salpêtrière, Paris, France; ^5^Translational Science and Experimental Medicine, Early RIA, BioPharmaceuticals R&D, AstraZeneca, Gothenburg, Sweden; ^6^Clinical Development—Respiratory Inhalation and Oral Development, Global Medicines Development, AstraZeneca, Gothenburg, Sweden; ^7^BD Biosciences, San Diego, CA, United States; ^8^Respiratory, Inflammation, and Autoimmunity, Medimmune, Gaithersburg, MD, United States; ^9^Sorbonne Université, Service d'Anatomie Pathologique, AP-HP Hôpital Pitié-Salpêtrière, Paris, France; ^10^Sorbonne Université, Unité Thyroïde Tumeurs Endocrines, AP-HP Hôpital Pitié-Salpêtrière, Paris, France

**Keywords:** thyroid autoimmune disease, regulatory T cells, follicular helper T cells, lymphoid tissue–inducer-like cells, Hashimoto's thyroiditis, Graves' disease

## Abstract

**Background:** Hashimoto's thyroiditis (HT) and Graves' disease (GD) are autoimmune thyroid disorders (AITDs). These conditions have been associated to abnormalities in circulating regulatory T cells (Tregs). We postulated that immune perturbations could be more pronounced at the thyroid tissue level.

**Methods:** The phenotype of PBMCs and immune cells infiltrating thyroid tissue from 19 patients with HT, 21 patients with GD, and 30 controls has been analyzed by flow cytometry.

**Results:** We report that blood and thyroid Treg cell subsets are similarly represented in all AITDs patients and controls. Increased Lymphoid tissue inducer (LTi)-like ILC3 and CXCR5^+^ PD-1^hi^ CD4^+^ T follicular helper cells (Tfh) tissue-infiltrating cells, together with the prevalence of tertiary lymphoid structures (TLS) and germinal centers (GCs) represented a typical immune signature in all HT and 60% of GD patients. In the remaining group of GD patients, the absence of the aforementioned abnormalities was associated with a higher prevalence of ophthalmopathy.

**Conclusion:** Tissue infiltrating Lymphoid Tissue inducer—like group 3 Innate Lymphoid cells and T follicular helper cells are increased in most thyroid autoimmune disease.

## Introduction

Hashimoto's thyroiditis (HT) and Graves' disease (GD) are autoimmune thyroid disorders (AITDs) that are characterized by the presence of specific autoantibodies. These autoantibodies are anti-thyroperoxidase and anti-thyroglobulin in HT, and thyroid stimulating hormone receptor (TRAb) autoantibodies, in GD, respectively. The immune mechanisms involved in the cellular- and humoral-mediated pathogenesis of these AITDs are not completely understood yet (1). Local lymphocytic infiltration is a common feature of HT but it also observed in GD (2).

Regulatory T (Treg) cells are crucial for the maintenance of self-tolerance. While the expression of the Forkhead Box P3 (FOXP3) transcription factor defines Treg cells in mice (3), similar FOXP3 expressing CD4^+^ T cells in humans are more heterogeneous. Our group has previously published data that classified these human Treg cells into (1) CD45RA^+^ FOXP3^lo^ naïve Treg (nTreg), and (2) CD45RA^−^ FOXP3^hi^ effector Treg (eTreg), both of which are suppressive and (3) the CD45RA^−^ FOXP3^lo^ non-suppressive CD4^+^ T cells (4). This is important as Treg cell deficiencies, either in number or function, can lead to autoimmune disorders such as rheumatoid arthritis (RA) or systemic lupus erythematous (SLE) (5–7). Moreover, there are also reports suggesting that Treg cells are defective either in number or function in AITDs. However, the molecular mechanisms accounting for these reported deficiencies have not been described yet (8–11).

The intrathyroidal pathology of patients with HT is characterized by lymphocytic infiltration and by the development of follicles resembling secondary lymphoid structures that are defined as tertiary lymphoid structures (TLS). Importantly, these intrathyroidal germinal centers (GCs) have been identified as producers of local anti-TPO autoantibodies (12). Therefore, one can expect in those structures the presence of CXC chemokine receptor 5 (CXCR5^+^) follicular helper T (Tfh) cells, that interact with B cells for antibody production (13) and of Lymphoid tissue inducer-like type 3 innate lymphoid (LTi-like ILC3) cells that are involved in the generation of lymphoid structures (14). While Tfh cells have been described in the peripheral circulation of patients with AITDs and in the thyroid of patients with GD (15, 16), the presence of ILC has not been described in AITD thyroid tissues yet.

Here, we show that whilst Treg cell compartments are not modified in the periphery or in the tissues, their LTi-like ILC3 and CXCR5^+^PD1^hi^ Tfh counterparts are highly prevalent in the thyroid of AITDs patients. We also show that the prevalence of ophthalmopathy correlates with the absence of infiltrating LTi-like ILC3s, CXCR5^+^PD1^hi^ Tfh cells and of local TLS structures.

## Materials and Methods

### Patients

Surgically-resected thyroid tissue was obtained from 19 HT patients (84% female; mean age ± SEM= 50 ± 3,2) and 21 GD patients (81% female; mean age ± SEM = 44 ± 3,7) (Table 1). The thyroid tissues used as a control were obtained from euthyroid glands (either around nodules or within goiter or on healthy lobes opposite to thyroid tumors) of 30 patients (73% female; mean age ± SEM = 52 ± 2,8). For some patients (seven per group), peripheral blood was also obtained at the same time as thyroid tissue biopsy. All patients gave informed consent. This study was approved by the institutional ethics committee (CPP Sud-Est VI registration number AU 1390, ID-RCB: 2017-A02871-52).

**Table 1:** Clinical characteristics of AITDs patients.

	**Controls (*n* = 30)**	**Hashimotos thyroiditis (*n* = 19)**	**Graves' diseases (*n* = 21)**
AGE (yr)	52 (20–76)	50 (23–73)	44 (19–82)
Gender (women/men)	22/8	16/3	17/4
TSH (mUI/1)	1.45 ± 0.15	9.42 ± 6.55	1.75 ± 0.54
Anti-thyroid treatment	0	0	21
Anti-TPO (UI/ml)	N/A	484.1 ± 206.1	N/A
Anti-Tg (UI/ml)	N/A	390.9 ± 111.4	N/A
TRAb (UI/1)	N/A	N/A	17.02 ± 3.83
**Infiltration**			
N/A	2	0	0
0	18	0	8
+	10	1	1
++	0	6	8
++/+++	0	8	3
+++	0	4	1

### Cells Isolation

Peripheral blood mononuclear cells (PBMCs) were separated by Ficoll-Hypaque density gradient centrifugation (Eurobio, Courtaboeuf, France). Infiltrating thyroid mononuclear cells (TMCs) were isolated from minced tissues by Ficoll–Hypaque centrifugation. PBMC and TMCs were then resuspended in RPMI 1640 (GIBCO—ThermoFisher scientific, Paisley, UK) supplemented with 10% fetal bovine serum (FBS). Cellular viability was assessed by trypan blue dye exclusion.

### Flow Cytometry Analysis

Freshly isolated PBMCs or TMCs were stained with anti-human antibodies; anti-hCD3 (-PerCP-Cy5.5 or BV510 or APC-H7; clone: SK7 or SP34-2), anti-hCD19 (-FITC; clone: 4G7), anti-hCD11b (-FITC; clone: ICRF44), anti-hCD45 (-APC-H7; clone: 2D1), anti-hCD127 (-BV510; clone: HIL7RM21), anti-hCD161 (-APC; clone: DX12), anti-hNkp44 (-PE; clone: 44.189; eBioscience), anti-hCRTH2 (-BV421; clone: BM16), anti-hCD117 (-PE-Cy7; clone: 104D2; eBioscience), anti-hCD4 (-PerCP-Cy5.5 or Pe-Cy7; clone: RPA-T4), anti-hCD45RA (-APC-H7 or FITC; clone: HI100), anti-hLAG3 (-PE; clone: T47-530), anti-hTIGIT (-Pe-Cy7; clone: MBSA43; eBioscience), anti-hPD1 (-BV510; clone: EH12.1), anti-hCXCR5 (-BV421; clone: RF8B2), and anti-hICOS (-PE; clone: DX29). All antibodies were obtained from BD Biosciences unless otherwise specified. Intracellular staining was performed on fixed and permeabilised cells (using the FOXP3 Transcription Factor Staining Buffer kit, eBioscience) with anti-hFOXP3 (-AF647 or PE; clone: 259D/C7) and anti-hBCL6 (-AF788; clone: K112-91). IL-2 production was detected after cells were stimulated with 1 μg/ml PMA and ionomycin in the presence of Golgi-Stop (BD biosciences) for 5 h. Stimulated cells were fixed and permeabilised and then stained with anti-hIL2 (-BV421; clone: MQ1-17H12). Cells viability was assessed using BD Horizon Fixable viability stain 780 (BD Biosciences). Data were acquired via the BD FACSCanto II Flow cytometer (BD biosciences) and analyzed with FlowJo v10 software (FlowJo, LLC).

### Histology Analysis

Lymphoid structures in thyroid tissues were studied in paraffin sections with haematoxylin-eosin saffron staining (HES).

### Statistical Analysis

All data are presented as Mean ± SE. Statistical comparisons were performed using the non-parametric Mann-Whitney *U*, Chi-squared or Spearman correlation tests. These were done using the GraphPad Prism software, V5.0 (GraphPad, San Diego). Data were considered significant when *p* < 0.05.

## Results

### Unaltered Circulating Treg Cell Subsets in AITDs

As abnormalities in Treg cells within the peripheral circulation have been described previously, we first studied these Treg subsets as defined by the expression of CD45RA and FOXP3 (Figure S1A) (4). We did this in patients with HT and GD and our controls were patients with no AITDs.

**Figure 1:**
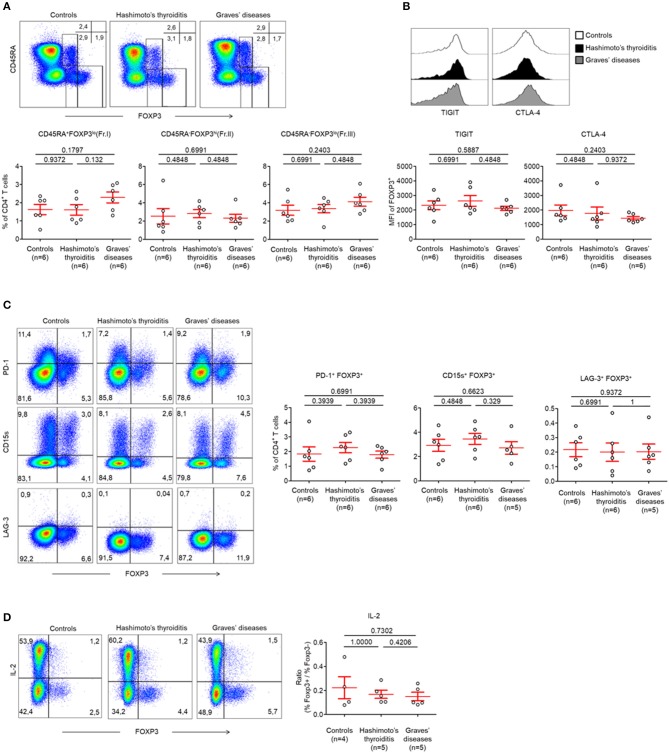
Normal circulating Treg cell compartments in AITDs. **(A)** Flow cytometry of FOXP3 expressing CD4^+^ T cells subsets defined by the expression of CD45RA and FOXP3 (top) and percent of CD45RA^+^ FOXP3^lo^ (Fraction I, Fr. I) nTreg, CD45RA^−^ FOXP3^hi^ (Fr. II) eTreg and CD45RA^−^ FOXP3^lo^ (Fr. III) T cells in peripheral blood of HT, GD and control patients (bottom). **(B)** Surface expression of TIGIT and intracellular expression of CTLA-4 by CD4^+^ FOXP3^+^ T cells in the peripheral blood of patients with HT, GD, and control patients. **(C)** Flow cytometry (left) and percent (right) of PD-1^+^ FOXP3^+^, CD15s^+^ FOXP3^+^, and LAG-3^+^ FOXP3^+^ cells among CD4^+^ T cells. **(D)** Flow cytometry (left) and percent (right) of IL-2 production by CD4^+^ T cells after stimulation with PMA and ionomycin for 5 h. Data shown in **(A–D)** are representative of the indicated number of independent experiments. Red bars represent mean ± SEM. Statistical comparisons were performed using the non-parametric Mann-Whitney test.

Between these groups, we identified no abnormalities in CD45RA^+^ FOXP3^lo^ (Fraction I; Fr. I) naïve Treg cells (nTreg) (Control patients: 1.62 ± 0.28%, HT: 1.61 ± 0.28%, GD: 2.29 ± 0.30%, *p* > 0.05) and CD45RA^−^ FOXP3^hi^ (Fraction II; Fr. II) effector Treg cells (eTreg) (Control patients: 2.53 ± 0.84%, HT: 2.82 ± 0.44%, GD: 2.31 ± 0.44%, *p* > 0.05) (Figure 1A). The proportion of CD45RA^−^ FOXP3^lo^ (Fraction III; Fr. III) non-Treg cells was also normal (Control patients: 3.18 ± 0.55%, HT: 3.37 ± 0.46%, GD: 4.12 ± 0.49%, *p* > 0.05) (Figure 1A).

Circulating FOXP3^+^ T cells of both groups of AITDs and control patients also displayed identical levels of Treg cell-related markers. This was evidenced by similar levels of *T-cell immunoreceptor with Ig and ITIM domains* (TIGIT) (MFI: Control patients: 2319 ± 301.1, HT: 2620 ± 363.7, GD: 2118 ± 140.9, *p* > 0.05), intracellular *Cytotoxic T-lymphocyte-associated protein 4* (CTLA-4) (MFI: Control patients: 1975 ± 367.6, HT: 1760 ± 436.8, GD: 1432 ± 117.2, *p* > 0.05), (Figure 1B, Figure S3A), *Programmed Death-1* (PD-1) (Control patients: 1.83 ± 0.48%, HT: 2.27 ± 0.35%, GD: 1.79 ± 0.24%, *p* > 0.05), CD15s [expressed on eTregs (17)] (Control patients: 2.93 ± 0.49%, HT: 3.45 ± 0.45%, GD: 2.71 ± 0.51%, *p* > 0.05) or *Lymphocyte-activation gene* (LAG-3) (Control patients: 0.22 ± 0.05%, HT: 0.20 ± 0.05%, GD: 0.20 ± 0.05%, *p* > 0.05) (Figure 1C). One key functional feature of Treg cells is their absence of production of interleukin-2 (IL-2) (18). We confirmed that Foxp3^low^ T cells have a low production of IL-2 whereas FOXP3^high^ eTreg cells did not produce IL-2 in AITDs patients or in controls. Hence, these cells display both phenotypic and functional characteristics of Treg cells (Figure 1D).

Taken together, these results indicate that, when FOXP3 expressing CD4^+^ T cells subsets are analyzed separately, the proportion of peripherally circulating Treg cells in AITDs patients is unaltered from those of controls (8, 19).

### Unaltered Thyroid Tissue Treg Cell Subsets in AITD

**Figure 2:**
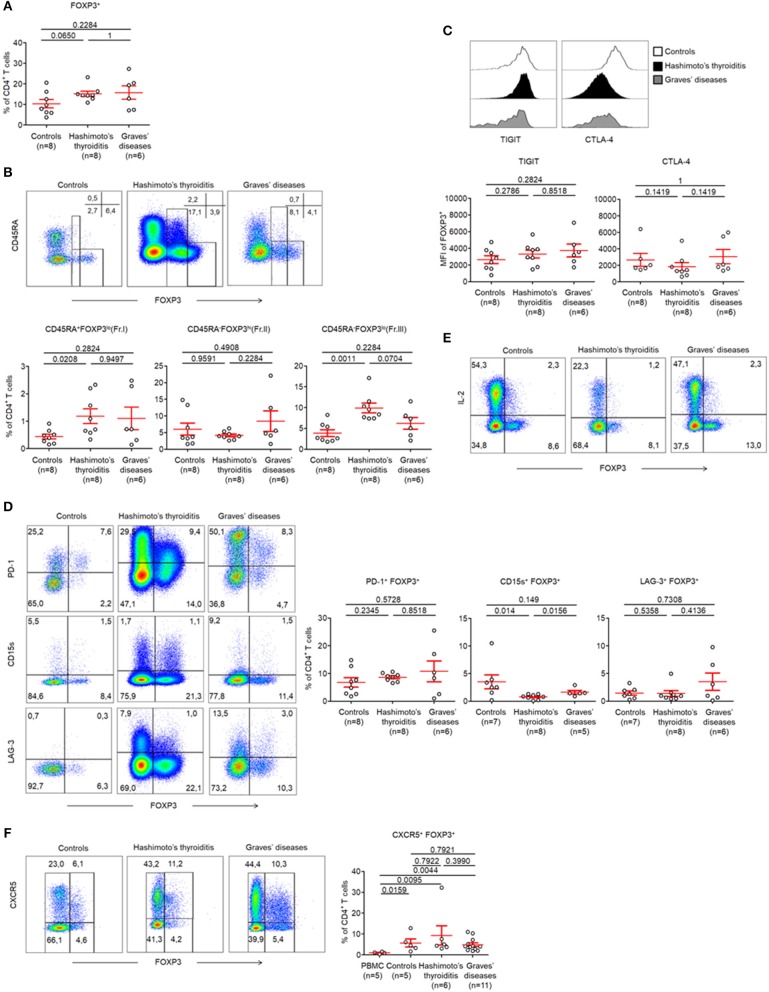
CD45RA^−^ FOXP3^lo^ (Fr. III) CD4^+^ T cells are increased in thyroid tissues of patients with AITDs. **(A)** Percent of CD4^+^ FOXP3^+^ T cells in thyroid tissues of HT, GD and control patients. **(B)** Flow cytometry of FOXP3^+^ subsets defined by the expression of CD45RA and FOXP3 (top) and percent (bottom) of CD45RA^+^ FOXP3^lo^ (Fr.I) nTreg, CD45RA^−^ FOXP3^hi^ (Fr. II) eTreg and CD45RA^−^ FOXP3^lo^ (Fr. III) T cells in thyroid tissues of HT, GD and control patients. **(C)** Surface expression of TIGIT and intracellular expression of CTLA-4 by CD4^+^ FOXP3^+^ T cells in thyroid tissues of HT, GD and control patients. **(D)** Flow cytometry (left) and percent (right) of PD-1^+^ FOXP3^+^, CD15s^+^ FOXP3^+^, and LAG-3^+^ FOXP3^+^ among CD4^+^ T cells. **(E)** Flow cytometry of IL-2 production by CD4^+^ T cells after stimulation with PMA and ionomycin for 5 h. Data shown are representative of experiments from 4 HT, 3 GD, and 7 control patients. **(F)** Flow cytometry (left) and percent (right) of CXCR5^+^ FOXP3^+^ CD4^+^ T cells in thyroid tissues of HT, GD and control patients. Data shown in **(B,C,E,F)** are representative of the indicated number of independent experiments. Red bars represent mean ± SEM. Statistical comparisons were performed using the non-parametric Mann-Whitney *U*-test.

As Treg cell subsets were unaltered in the peripheral circulation, we next decided to investigate intrathyroidal FOXP3^+^ CD4^+^ T cell subsets (Figure S1B). We found that the proportions of intrathyroidal FOXP3 expressing cells among CD4^+^ T cells were unaltered in both controls and AITDs patients (Control patients: 10.34 ± 2.03%, HT: 15.23 ± 1.28, GD: 15.76 ± 3.27%, *p* > 0.05) (Figure 2A). We also found no changes between these groups with regards to infiltrating CD45RA^−^ FOXP3^hi^ (Fr. II) eTreg cells (Control patients: 6.03 ± 1.83%, HT: 4.14 ± 0.43, GD: 8.42 ± 3.11%, *p* > 0.05) (Figure 2B). We found that infiltrating CD45RA^+^ FOXP3^lo^ (Fr. I) nTreg cells (Control patients: 0.44 ± 0.01%, HT: 1.18 ± 0.27, *p* < 0.05) and infiltrating CD45RA^−^ FOXP3^lo^ (Fr. III) non-Treg cells were increased in the HT group (9.91 ± 1.15 vs. 3.86 ± 0.83%, *p* < 0.01) but not in GD (Figure 2B).

Within FOXP3^+^ CD4^+^ T cells, we found no changes in the expression levels of TIGIT (MFI: Control patients: 2,637 ± 464.3, HT: 3,304 ± 478.6, GD: 3,730 ± 765.4, *p* > 0.05) and of CTLA-4 (MFI: Control patients: 2,662 ± 768.3, HT: 1,843 ± 494.0, GD: 3,055 ± 856.2, *p* > 0.05) (Figure 2, Figure S3B). These cells also demonstrated unaltered levels of PD-1 (Control patients: 675 ± 1.70%, HT: 8.64 ± 0.52%, GD: 10.79 ± 3.76%, *p* > 0.05) and LAG-3 (Control patients: 1.47 ± 0.38%, HT: 1.38 ± 0.53%, GD: 3.5 ± 1.57%, *p* > 0.05). However, we identified reduced CD15s expression in HT tissue when compared to GD and control patients (0.82 ± 0.15 vs. 0.79 ± 0.16% and 1.64 ± 0.33%, respectively, *p* < 0.05) (Figure 2D). Like peripheral cells, we showed that intrathyroidal FOXP3^high^ eTreg cells did not produce IL-2 in AITDs patients or in controls (Figure 2E). Put together, our results do not support abnormalities in infiltrating intrathyroidal Treg cells. Our results also indicate that the Treg compartments are unaltered in the peripheral circulation and thyroids of AITDs patients.

Finally, one subset of FOXP3 expressing cells that is included in the FOXP3^low^ subset is the T follicular regulatory (Tfr) cell subset (characterized by expression of CXCR5) (20). We confirmed the presence of these Tfr cells in the thyroid tissues of controls, HT and GD without significant differences in their prevalences (Figure 2F).

### Thyroid-Infiltrating LTi-Like ILC3 Cells in AITD

**Figure 3:**
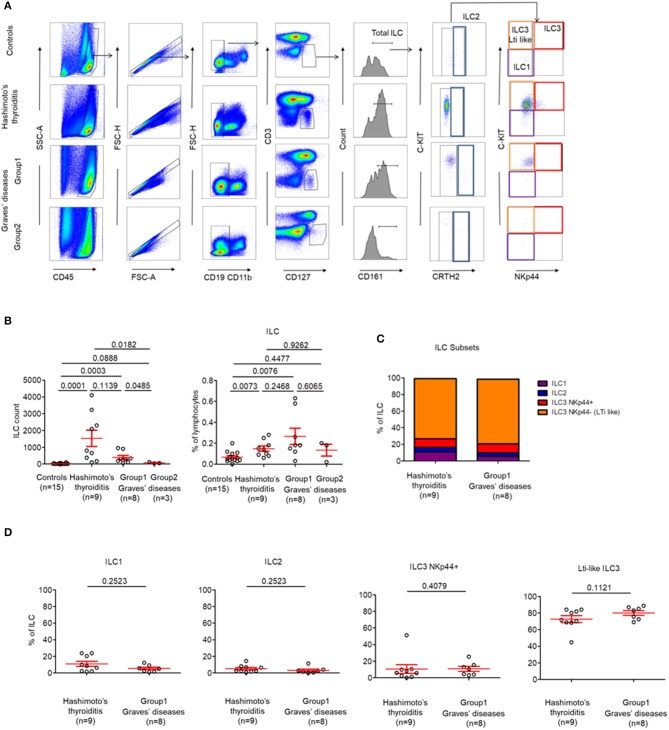
LTi-like ILC3 cells are highly prevalent in the thyroid tissues of patients with HT and the majority of patients with GD. **(A)** Flow cytometry of ILC subsets in thyroid tissues of HT, GD and control patients. Data shown are representative of the indicated number of independent experiments. **(B)** Percent of total ILC among lymphocytes in thyroid tissues of HT, GD, and control patients (left) and ILC count (right). **(C)** Distribution of ILC1, ILC2, ILC3 NKp44^+^, and ILC3 NKp44^−^ (LTi like) among ILC in the thyroid tissues of HT, GD group 1 patients. **(D)** Percent of ILC1, ILC2, ILC3 NKp44+, and ILC3 NKp44- (LTi like) among ILC in the thyroid tissues of HT, GD group1 patients. Red bars represent mean ± SEM. Statistical comparisons were performed using the non-parametric Mann-Whitney test. In **(C,D)**, HT and GD group 2 patients are not shown because of the absence of ILC in tissues.

Due to the intrathyroidal presence of Tfr cells, we hypothesized that other immune cells that are usually involved in lymphoid development such as LTi-like cells or follicular T cells could also be present within the thyroid (21). Thus, we decided to investigate for the presence of infiltrating ILC subpopulations in patients with AITDs with a particular focus on LTi-like ILC3 cells, which are involved in the development of secondary and tertiary lymphoid organs (14). We identified that globally-infiltrating ILCs (CD3^−^ CD4^−^ CD19^−^ CD11b^−^ CD45^+^ CD127^+^ CD161^+^) represented a very small part of intrathyroidal lymphocytes (0,14 ± 0.02%) in most of AITDs (Figures 3A,B). Indeed, while in controls and a group of GD (Group2, *n* = 3 out of 11, 27%), ILCs could not be observed in tissues, we observed a high proportion of LTi-like ILC3 (CRTH2^−^ C-kit^+^ NKp44^−^) among infiltrating ILCs in the tissues of all HT patients and another group of GD patients (Group1, *n* = 8 out of 11, 83%) (Figure 3C). In the latter groups, the proportions of ILC1, ILC2, and ILC3 NKp44^+^ were identical (Figure 3D). Therefore, two groups of GD patients can be defined based on the presence of a majority of infiltrating LTi-like ILC3 cells within ILCs: Group 1 (with proportion of 78.16 ± 3.22% among whole ILCs) and Group 2 (where ILC are not observed). Importantly, this finding is tissue-specific as the distributions of ILCs in the circulation was normal (Figure S2). Altogether, our results indicate that LTi-like ILC3 are increased in tissues of all patients with HT and a majority of patients with GD. This also suggests the intrathyroidal presence of TLS.

### Thyroid GCs and Tfh Cells in AITD

**Figure 4:**
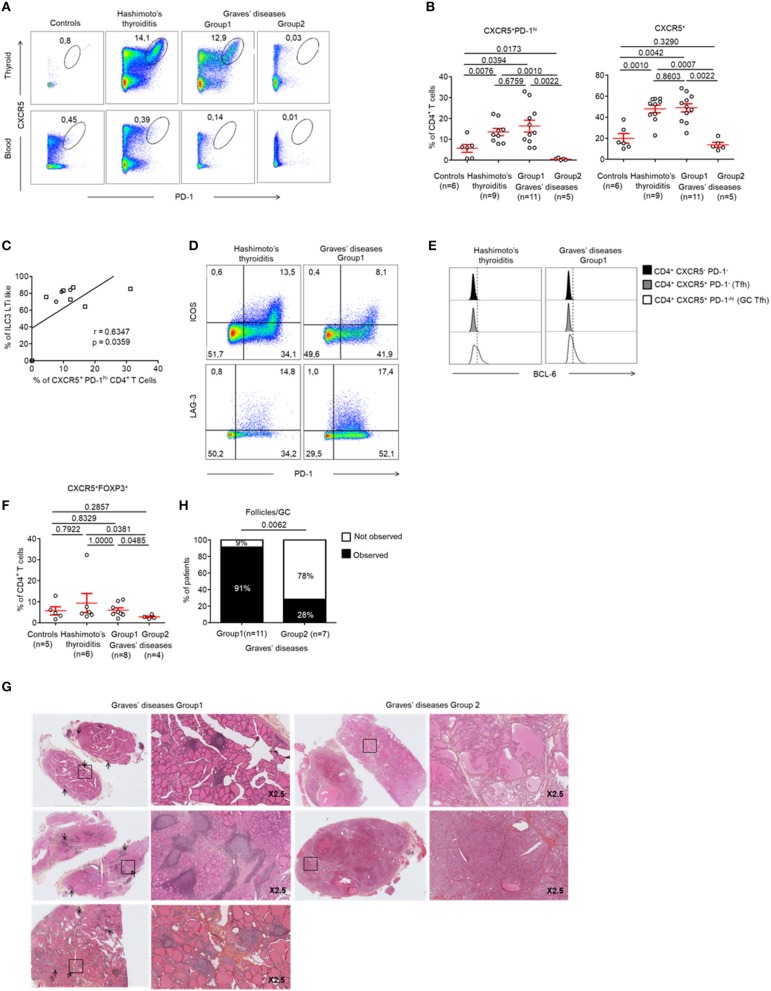
Development of GCs and prevalence of Tfh cells in the thyroid of patients with HT and a majority of patients with GD. **(A)** Flow cytometry analysis of the expression CXCR5 and PD-1 expression by CD4^+^ T cells in the thyroid tissue (top) and blood (bottom) of patients with HT, GD, and control patients. **(B)** Percent of FOXP3^−^CXCR5^+^ PD-1^hi^ CD4^+^ (left) and FOXP3^−^CXCR5^+^ CD4^+^ T cells (right) among CD4^+^ T cells in thyroid tissues of patients with HT, GD and control patients. **(C)** Correlation between LTi-like ILC3 and CXCR5+ PD1^hi^ CD4^+^ T cells prevalence in the thyroid tissues of patients with GD. **(D)** Expression of PD-1 and ICOS (top) or LAG-3 (bottom) by CXCR5^+^ CD4^+^ T cells. Data shown are representative of 4 HT and 3 GD experiments. **(E)** Intracellular BCL6 expression by CXCR5^−^ PD-1^−^ CD4^+^, CXCR5^+^ PD-1^−^ CD4^+^ (Tfh) and CXCR5^+^ PD-1^hi^ CD4^+^ (germinal center T follicular helper) cells. Data shown are representative of 6 HT and 3 GD experiments. **(F)** Prevalence of CXCR5^+^ FOXP3^+^ CD4^+^ T cells in thyroid tissues among CD4^+^ T cells of HT, GD, and control patients. **(G)** Histologic sections (HES staining) of thyroid tissues of GD patients. Lymphocytic infiltration and GC are shown with arrows. **(H)** Contingency analysis: percent of patients with or without GC among GD patients. Red bars represent mean ± SEM. Statistical comparisons were performed by non-parametric Mann-Whitney *U*-test, Chi-squared test and Spearman correlation coefficient test.

Tfh cells are involved in the development of TLS. We found increased Tfh cells [defined as CXCR5^+^ PD-1^hi^ CD4^+^ (22)] in the GCs of all patients with HT when compared to controls (13.48 ± 5.27 vs. 5.64 ± 4.65%, respectively, *p* < 0.05) (Figures 4A top, B). In contrast, this population was not present in the peripheral blood (Figure 4A, bottom). Most importantly, we found that Tfh cells were increased specifically in the tissues of patients from GD Group 1 who demonstrated increased LTi-like ILC3 cells too. This finding was not made in patients from GD Group 2. Importantly, we could determine that the prevalence of CXCR5^+^ PD-1^hi^ CD4^+^ Tfh cells increased with the prevalence of LTi-like ILC3 cells (*r* = 0.63, *p* = 0.036) (Figure 4C). The infiltrating CXCR5^+^ PD-1^hi^ CD4^+^ Tfh cells displayed an activated phenotype that is usually observed in GCs. This was defined as the high expression of *inducible T-cell costimulator* (ICOS), LAG-3 and *B-cell lymphoma protein* 6 (BCL-6) (Figures 4D,E). Of note, the two groups of GD patients also displayed differences in the prevalence of CXCR5^+^ FOXP3^+^ Tfr cells. These cells were significantly decreased in Group 2 (5.98 ± 1.14 vs. 2.75 ± 0.46% *p* < 0.05) (Figure 4F). Therefore, GD Group 1 and Group 2 can be defined by tissue infiltration of both LTi-like ILC3 cells and Tfh cells. We could further confirm these results histologically. As expected, no follicles were observed in the thyroid tissues of GD patients from Group 2 who did not display CXCR5^+^ PD-1^hi^ CD4^+^ Tfh or LTi-like ILC3 cells (Figures 4G,H).

### Ophthalmopathy Mostly Occurs in the Absence of TLS in Thyroid Tissue

**Figure 5:**
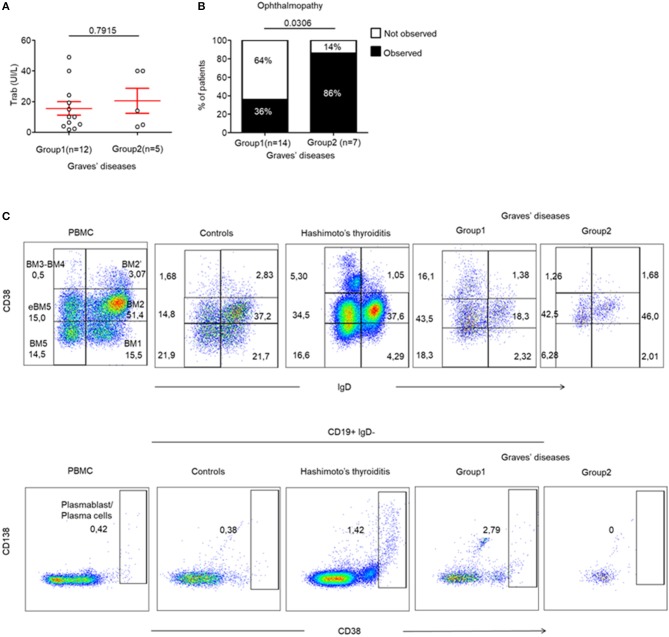
Ophthalmopathy mostly occurs in the absence of TLS in thyroid tissue. **(A)** TRAb titers before surgery in GD patients. **(B)** Contingency analysis: percent of patients with or without the occurrence of ophthalmopathy in patients of GD. Red bars represent mean ± SEM. Statistical comparisons were performed by non-parametric Mann-Whitney test and Chi-squared test. **(C)** Flow cytometry of infiltrating B cell subsets [BM1-BM2: naïve B cells; BM2': germinal center founder B cells; BM3-BM4: germinal center B cells (centroblast—centrocyte); BM5: memory B cells; eBM5: early memory B cells] in the thyroid of a patient with control, HT, GD group 1 and GD group 2 (top). Antibodies producing B cells are characterized by the CD38^++^ phenotype (bottom). Results are representative of 3 HT, 3 GD Group1, 2 GD Group2, and 2 CTRL thyroid tissues.

Finally, we wanted to identify whether the presence of infiltrating Tfr, Tfh and LTi-like ILC3 cells could be correlated with specific humoral or clinical phenotypes of AITDs. This was important as these cells are involved in TLS and in the formation of GCs, which leads to antibody production by B cells. Therefore, we investigated whether the production of pathogenic anti-TRAb could be affected by the differences observed in the 2 GD patient subgroups. In fact, we found that TRAb levels were similar in both groups of GD patients (15.57 ± 4.4 vs. 20.50 ± 8.16, *p* = 0.7915) (Figure 5A). In addition, the titres of TRAb antibodies did not correlate with the percentage of CXCR5^+^ PD-1^hi^ CD4^+^ Tfh cells *(data not shown)*. However, we clearly identified that 86% (6/7) of GD patients without thyroid follicles (Group 2) developed an ophthalmopathy while only 36% (5/14) of GD patients with thyroid follicles (Group 1) did (*p* = 0.0306) (Figure 5B), but we did not find difference in term of severity between group 1 and 2 (Table S1). In a limited number of tissue samples, we could also analyse tissue B cells and could observe in HT and GD group 1 thyroid the presence of germinal center B cells also called mature B cell population 3 and 4 (BM 4 and 4) that are defined by the CD38^+^IgD^−^ phenotype (23) (Figure 5C, top) and antibodies producing CD38^++^ that included CD138^+^ plasma cells and CD138^−^ plasmablast, but not in Control and GD group 2 (Figure 5C, bottom). Putting all these data together, they indicate that the occurrence of ophthalmopathy may be independent of dysregulated local immune responses in thyroid.

## Discussion

We have provided here for the first time a comprehensive view of tissue immunoregulatory mechanisms that are involved either in the prevention or the induction of organ specific autoimmunity in the thyroid.

First, we have demonstrated that there were no alterations in the quantities of circulating Treg cell subsets in patients with AITDs. This finding is in contrast to previous observations that have shown an increase in circulating Treg cells in AITDs (8, 24) or a decrease (10, 19). These findings can be reconciled by the use of different phenotypic markers to define/classify Treg cell subsets. Several functionally different subsets have been defined in FOXP3 expressing CD4^+^ T cells (4, 25) and importantly, not all FOXP3 expressing cells are regulatory in humans. These suppressive and non-suppressive subsets can be precisely differentiated by surface markers such as those utilized in this study and by others (4, 26, 27). We also showed that circulating Treg cells were functionally comparable to those of healthy donors, as they do not produce IL-2. Indeed, the absence of production of IL-2 is a characteristic feature of Treg cells. In order to facilitate their suppressive function, their other features include expression of CD25, CTLA-4, TIGIT as well as expression of FOXP3 (4, 18, 28). We demonstrated an equivalent expression of CTLA-4 and TIGIT in all circulating FOXP3^+^ T cells subpopulation in AITDs and controls, with a higher expression in FOXP3^high^ eTreg cells. However, contrary to our findings, a deficiency in Treg cell suppressive function has indeed been described in AITDs (10, 11). That observation can be explained by the fact that, in their study, Treg cells were analyzed as a homogeneous population defined by the CD4^+^C25^+^ or CD4^+^CD25^high^ phenotype. Hence, their observed decrease in Treg suppressive function could be due to the dilution of fully functional Treg cells with activated cells that are not suppressive (5).

Additionally, we also studied infiltrating Treg cells in the thyroids of patients with AITDs and again, did not observe abnormalities in their Treg cell subsets, the highest proportion of CD45RA^+^ FOXP3^lo^ (Fr. I) nTreg cells explained by patients ages (Control mean age = 58, HT mean age = 42, GD mean age 47). It should be noted that in this present study, the number of infiltrating cells especially in controls is a limiting factor for setting up suppression function assay. Due to the absence of such abnormalities in both the periphery and thyroids of patients with AITDs, we then focused on other types of immune cells involved in local tissue immunoregulation that is ILCs and Tfh cells.

We found that ILCs were barely detectable in the peripheral blood of controls and patients with AITDs. However, we observed a consistent infiltration of ILCs in the thyroids of all patients with HT and of a majority of patients with GD. In comparison, we detected no ILCs in the control thyroids. Importantly, this infiltration was exclusively due to the prevalence of LTi-like ILC3 with the NKp44 negative phenotype. These cells play a major role in the development of lymphoid organs through their production of chemokine-like LTα1β2 that is essential for organ development (29, 30). Of note, the predominant subsets within the circulating ILCs were ILC2, as it observed in controls (31). This indicates that thyroid specific ILCs abnormalities based on the polarization of ILCs responses toward LTi-like ILC3 responses are occurring in most thyroid autoimmune diseases.

Furthermore, our findings were similar when analyzing Tfh cells. The CXCR5^+^ PD-1^hi^ CD4^+^ activated Tfh cells were only observed in the thyroids of all patients with HT and of a majority of patients with GD. However, these cells were barely detectable in control thyroids and the peripheral blood of both controls and AITDs patients.

Interestingly, the GD patients from Group 1 that demonstrated high tissue infiltration with LTi-like ILC3 cells also demonstrated high infiltration of activated Tfh cells. More specifically, the percentage of tissue-infiltrating activated Tfh cells highly correlated with the extent of tissue infiltrating LTi-like ILCs. Together, these findings indicate a close relationship between these immune cell subsets (21).

Within the follicular T cell compartments, the CXCR5^+^ FOXP3^+^ Tfr cells have been described as regulators of other Tfh and B cells, subsequent indirect modulators of GC responses and the local production of antibodies (20). In patients with Sjogren's syndrome or RA, autoantibody titres are known to correlate with the Tfr/Tfh ratio in the peripheral blood. However, we did not observe such a correlation in AITDs—both GD and HT *(data not shown)*. Similarly, we did not observe any difference in the prevalence of tissue infiltrating Tfr cells in both HT and GD when compared to control thyroids. However, the prevalence of Tfr cells was slightly higher in both HT and GD Group 1 when compared to Group 2. Put together, all these findings are in favor of the absence of a significant role of Tfr cells in the pathogenesis of AITDs.

Whilst the prevalence of tissue infiltrating LTi-like ILC3 cells has not been reported in both HT and GD yet, the infiltration of thyroid tissues by Tfh has indeed been reported in GD (16, 32). In their studies, Zhang et al. and Aust et al. demonstrated intrathyroidal infiltration of Tfh cells as well as the presence of CXCR5, *CXC chemokine ligand 13* (CXCL13) and IL-21 within the tissues of patients with AITDs. However, they were unable to classify their GD patients into two distinct subsets. Similarly, another study also demonstrated an increase in circulating activated Tfh cells in AITDs (15). Those cells were also phenotypically defined as CD4^+^CXCR5^+^PD-1^high^ cells. Put together, CD4^+^CXCR5^+^PD-1^high^ activated Tfh cells could be clearly distinguished in the thyroid tissue but were virtually absent, in our experience, in both control thyroids and the blood of AITDs patients. Therefore, we consider that peripherally activated Tfh cells can marginally contribute toward the local autoimmune responses in AITDs.

Importantly, we demonstrated a significant positive correlation between Tfh and LTi-like ILC3 cells. This is a key finding as both populations are involved in the development of tertiary lymphoid organs and of GCs (21). These GCs are involved in the production of local autoantibodies in AITDs. Moreover, the binding of TPO and Tg within the thyroid TLS of AITDs patients favors the local presence/production of anti-TPO and anti-Tg autoantibodies (12). Indeed, whilst GCs were present in the thyroids of all HT patients and almost all GD Group 1 patients, they were absent in most of GD Group 2 patients. Morevover, we also observed in a limited number of analyzed samples infiltrating antibodies producing B cells in HT and GD group 1 tissues while those were absent in control and Group 2 GD tissues. Even if no correlation was observed between TRAb levels and the percentage of CXCR5^+^ PD-1^hi^ CD4^+^cells or the presence of GCs, we observed that GD Group 2 patients who did not develop local thyroid TLS and GCs were more likely to develop ophthalmopathy than GD Group 1 patients. Based on this and because the evolution of ophthalmopathy is often independent of the evolution of the thyroid disease (33, 34), we hypothesize that:

1. Intrathyroidal immune abnormalities as observed in patients with HT and from GD Group 1 lead to the development of local TLS and of GCs that produce autoantibodies which, are responsible (at least in GD) for local autoimmune pathology—but not ophthalmopathy.
2. Similar abnormal immune responses in orbital tissue could lead to the development of TLS and GCs; thus facilitating ophthalmopathy and/or thyroid disease. Unfortunately, we could not verify this hypothesis as biopsies are contra-indicated in patients with active ophthalmopathy (35).

In this paper, we have demonstrated that patients with HT have a common pattern of abnormal local immune responses. These include; increased CXCR5^+^ FOXP3^+^ Tfr, LTi-like ILC3, and CXCR5^+^ PD-1^hi^ CD4^+^ Tfh cells (but normal Treg cell subsets). They likewise have an increased prevalence of TLS and GCs. We could not determine whether these two patterns described two distinct forms of GD or an evolutionary form of the diseases. Indeed, clinical history of patients with GD indicates that there is no differences in the duration of disease between Group 1 and 2. In addition, we can exclude here a potential immunomodulatory effect of anti-thyroid drugs in Group 2 patients (36) because (1) some GD group 1 patients that are efficiently treated with anti-thyroid drugs, i.e., they are euthyroid, display an increased prevalence of TLS and GCs with increased CXCR5^+^ FOXP3^+^ Tfr, LTi-like ILC3, and CXCR5^+^ PD-1^hi^ CD4^+^ Tfh cells and (2) some group 2 patients are still displaying hyperthyroidism without infiltrates while under treatment (Table S1).

We also demonstrated that GD is characterized by two different patterns of local immune responses: one that is similar to that observed in HT with a weak prevalence of ophthalmopathy, and another without specific immune cell and lymphoid abnormalities involving a higher prevalence of ophthalmopathy. How such abnormalities are provoked remains to be determined.

## Data Availability Statement

All datasets generated for this study are included in the article/Supplementary Material.

## Ethics Statement

The studies involving human participants were reviewed and approved by CPP Sud-Est VI registration number AU 1390, ID-RCB: 2017-A02871-52. The patients/participants provided their written informed consent to participate in this study.

## Author Contributions

All authors listed have made a substantial, direct and intellectual contribution to the work, and approved it for publication.

### Conflict of Interest

RM and GM were employed by the company Astrazeneca. RB was employed by the company BD bioscience. OV was employed by the company MedImmune. The remaining authors declare that the research was conducted in the absence of any commercial or financial relationships that could be construed as a potential conflict of interest.

## Supplementary Material

Figure S1 Gating strategy for CD4^+^ T cells analysis. **(A)** Representative gating strategy of flow cytometry of CD4^+^ T cells subsets in the blood for HT, GD and control patients. **(B)** Representative gating strategy of flow cytometry of CD4^+^ T cells subsets in the thyroid for HT, GD and control patients.

Figure S2 Normal peripheral ILC distribution in AITDs. **(A)** Flow cytometry of ILC subsets in the blood of HT, GD and control patients. Data shown are representative of the indicated number of independent experiments. **(B)** Percent of total ILC among lymphocytes in the blood of HT, GD, and control patients. **(C)** Distribution of ILC1, ILC2, ILC3 NKp44^+^, and ILC3 NKp44^−^ (LTi like) among ILC in the blood of HT, GD and control patients. Red bars represent mean ± SEM. Statistical comparisons were performed using the non-parametric Mann-Whitney test.

Figure S3 Normal expression of TIGIT and CTLA-4 in peripheral and infiltrating Foxp3+ T cells subsets. **(A)** Surface expression of TIGIT and intracellular expression of CTLA-4 by CD4^+^ FOXP3^+^ T cells subsets in the peripheral blood of patients with HT, GD and control patients. **(B)** Surface expression of TIGIT and intracellular expression of CTLA-4 by CD4^+^ FOXP3^+^ T cells subset in thyroid tissues of HT, GD, and control patients. Red bars represent mean ± SEM. Statistical comparisons were performed using the non-parametric Mann-Whitney test.

Table S1 Clinical comparison between GD patient groups.
