# Supplementary figures and images for: Tissue Infiltrating LTi—Like Group 3 Innate Lymphoid Cells and T Follicular Helper Cells in Graves' and Hashimoto's Thyroiditis
Source: Front Immunol. 2020 Apr 9;11:601. doi: 10.3389/fimmu.2020.00601 (PMC7160246; doi:10.3389/fimmu.2020.00601)

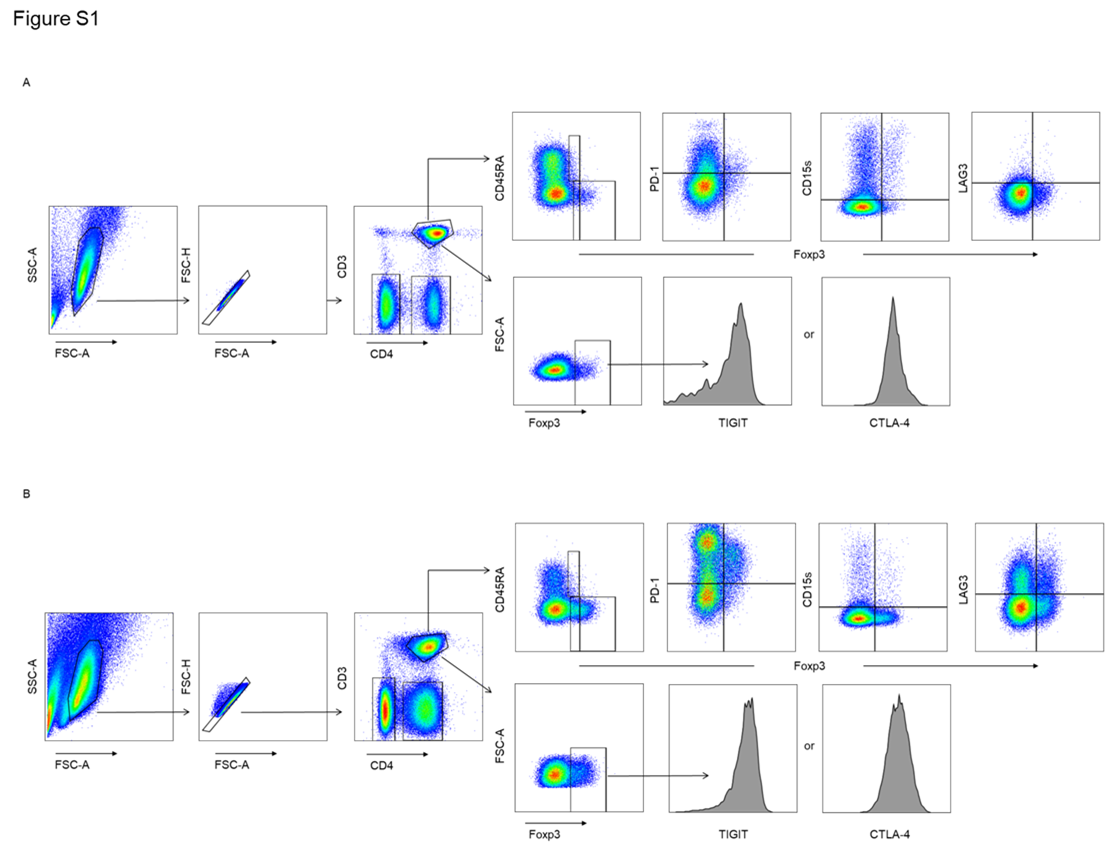

Supplement: Figure S1 — Gating strategy for CD4+ T cells analysis. (A) Representative gating strategy of flow cytometry of CD4+ T cells subsets in the blood for HT, GD and control patients. (B) Representative gating strategy of flow cytometry of CD4+ T cells subsets in the thyroid for HT, GD and control patients. [file Image_1.TIF]

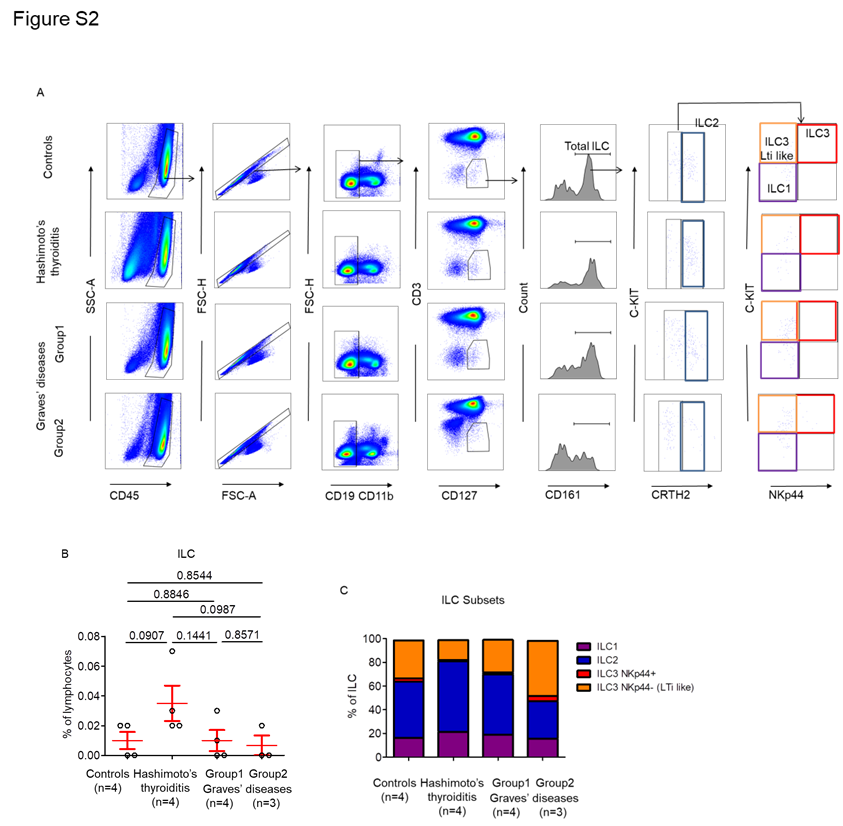

Supplement: Figure S2 — Normal peripheral ILC distribution in AITDs. (A) Flow cytometry of ILC subsets in the blood of HT, GD and control patients. Data shown are representative of the indicated number of independent experiments. (B) Percent of total ILC among lymphocytes in the blood of HT, GD, and control patients. (C) Distribution of ILC1, ILC2, ILC3 NKp44+, and ILC3 NKp44− (LTi like) among ILC in the blood of HT, GD and control patients. Red bars represent mean ± SEM. Statistical comparisons were performed using the non-parametric Mann-Whitney test. [file Image_2.tif]

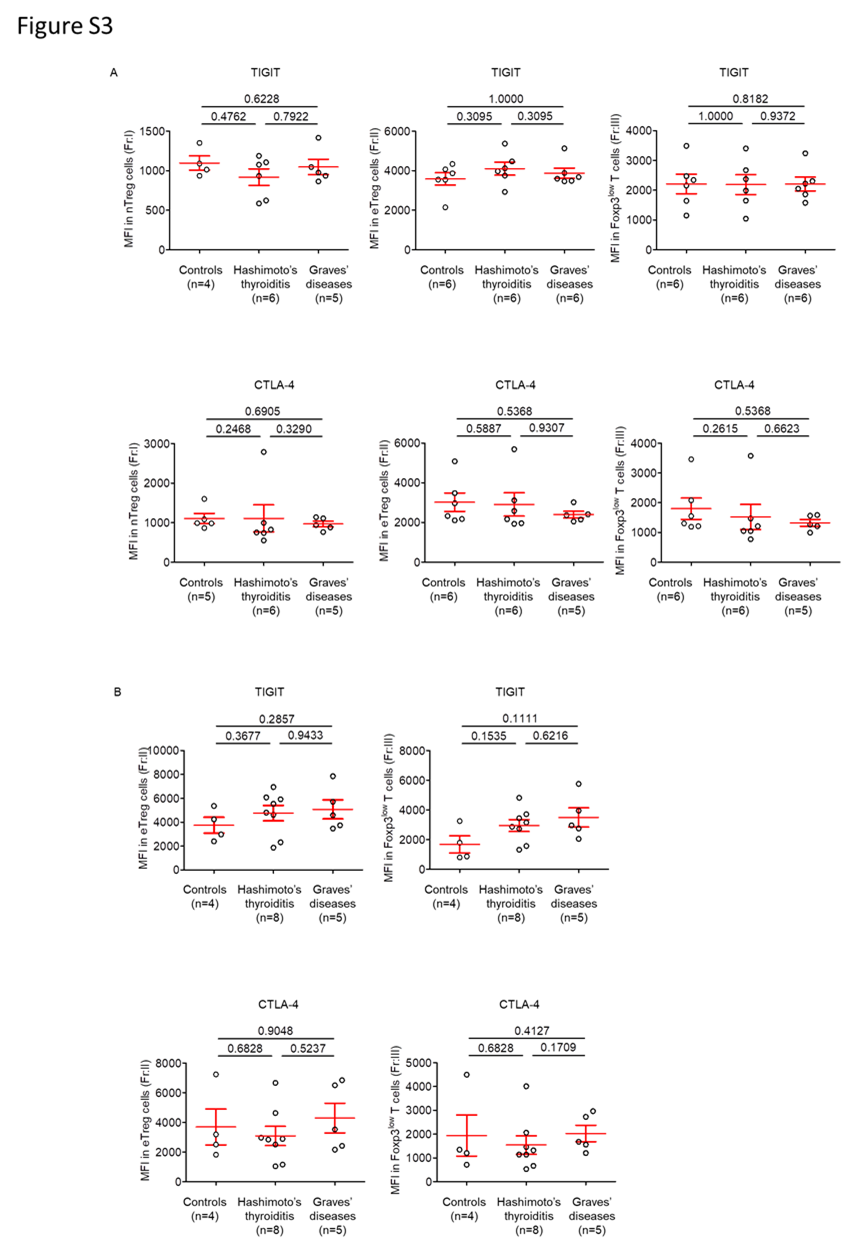

Supplement: Figure S3 — Normal expression of TIGIT and CTLA-4 in peripheral and infiltrating Foxp3+ T cells subsets. (A) Surface expression of TIGIT and intracellular expression of CTLA-4 by CD4+ FOXP3+ T cells subsets in the peripheral blood of patients with HT, GD and control patients. (B) Surface expression of TIGIT and intracellular expression of CTLA-4 by CD4+ FOXP3+ T cells subset in thyroid tissues of HT, GD, and control patients. Red bars represent mean ± SEM. Statistical comparisons were performed using the non-parametric Mann-Whitney test. [file Image_3.tif]

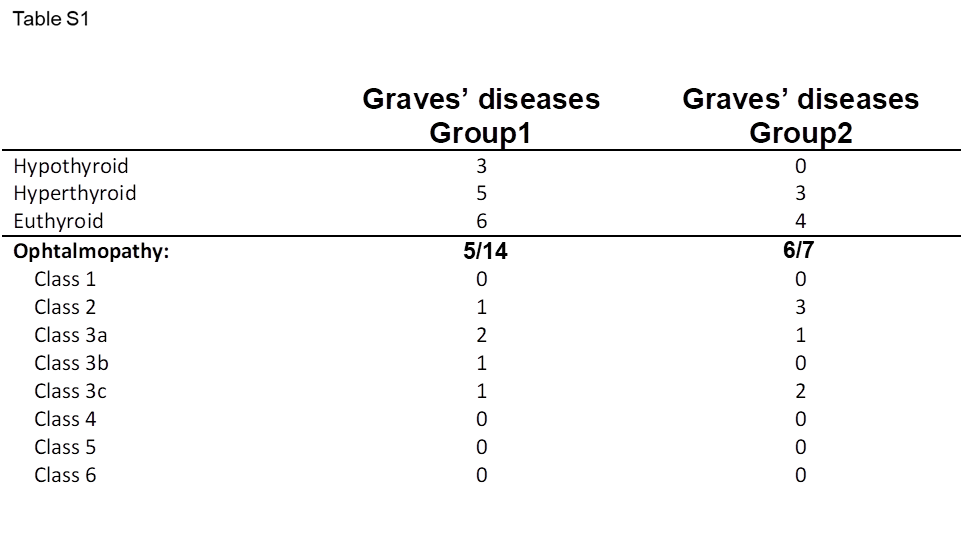

Supplement: Table S1 — Clinical comparison between GD patient groups. [file Image_4.TIF]
